# Term Infant with Cerebral Venous Sinus Thrombosis

**DOI:** 10.1155/2020/8883007

**Published:** 2020-09-18

**Authors:** Sanket Jani, Ralph Ariss, Pradeep Velumula, Deniz Altinok, Sanjay Chawla

**Affiliations:** ^1^Department of Pediatrics, Division of Neonatology, Central Michigan University School of Medicine, Detroit, MI, USA; ^2^Neonatology Perinatology Fellowship Program, Children's Hospital of Michigan, Detroit, MI, USA; ^3^Department of Pediatric Radiology, Children's Hospital of Michigan, Detroit, MI, USA

## Abstract

Hypernatremic dehydration in neonates is a common condition in an exclusively breastfed infant but often underdiagnosed. Any newborn who has lost more than 10% of birthweight should be carefully evaluated and monitored for clinical features of dehydration. Efforts such as frequent follow-up for weight check, and formula supplementation, if needed, should be provided to a neonate at risk of developing complications of dehydration. Adequate lactation consultation, both inpatient and outpatient, should also be provided, especially to the primigravida mother. Here, we present a case of a neonate with severe hypernatremic dehydration caused by inadequate lactation in a primigravida mother, which resulted in cerebral venous sinus thrombosis leading to significant intracerebral hemorrhage. The infant suffered permanent neurologic damage and was sent home on technological devices (tracheostomy and gastrostomy tubes). Further, we provide a brief review of hypernatremic dehydration and sinus venous thrombosis in neonates.

## 1. Case Report

A 7-day-old female infant was brought to the emergency department with decreased responsiveness and poor feeding. She was born at 38^4/7^ weeks' gestation, with a birthweight of 2770 g (10^th^–25^th^ percentile) to a 38-year-old primiparous woman with adequate prenatal care. The pregnancy was complicated by gestational thrombocytopenia diagnosed in the third trimester, which required treatment with hydrocortisone. Her prenatal laboratory findings, including syphilis rapid plasma reagin, hepatitis Bs antigen, HIV, gonorrhea, and chlamydia, were negative. The result of her group B *Streptococcus* screening test was positive. An urgent cesarean delivery was performed because of her nonreactive nonstress test at the obstetric clinic. Rupture of amniotic membranes occurred at delivery. The postdelivery course of infant was uncomplicated; the Apgar scores were 7 and 9 at 1 and 5 minutes, respectively. In the postnatal ward, the neonate fed poorly, with low maternal milk supply. The mother was seen by a lactation consultant who advised her to continue breastfeeding. The neonate was discharged from the hospital 48 hours after birth with a weight of 2,665 g (4% loss from birthweight), and the family advised to arrange follow-up with the primary care physician. The infant's platelet count, obtained before discharge because of the maternal history of gestational thrombocytopenia, was noted to be 180,000/*μ*L (180 × 10^9^/L). At the primary care physician visit on day 3 of age, the infant's weight was 2,475 g (∼11% lower than birthweight). She was noted to be otherwise well appearing though the mother continued to report difficulty with latching and breast milk supply. She received lactation consultation again in the office.

On day 6, the infant became increasingly lethargic and uninterested in feeding. She was taken to the emergency department (ED) where it was recorded that she had an average of 3 wet diapers/day and less than 1 stool/day over the past 2 days. In addition, the mother endorsed a history of right-sided gaze and head deviation for a few seconds while the infant was being placed in the car seat on the way to the ED. On initial physical examination at the ED, the infant was lethargic and minimally responsive, with a prolonged capillary refill time and appeared grossly dehydrated with standing skinfolds. Her vital signs were as follows: temperature, 34.2°C (93.5°F); heart rate, 184 beats/min; respiratory rate, 64 breaths/min; and oxygen saturation, 100% in room air. Her weight was 2,220 g (∼20% below birthweight). Multiple short self-limiting brief episodes of right gaze deviation and left arm tonic-clonic movements were also noted. Laboratory testing in the ED revealed the following.

### 1.1. Electrolytes

  Sodium 173 mEq/L (reference range 135–145 mEq/L)
  Potassium 6.8 mEq/L (reference range 3.8–6.0 mEq/L)
  Chloride 130 mEq/L (reference range 95–110 mEq/L)
  BUN 165 mg/dL (reference range 7–25 mg/dL)
  Creatinine 1.92 mg/dL (reference range 0.2–0.4 mg/dL)
  Bicarbonate 10 mEq/L (reference range 18–24 mEq/L)
  Glucose 111 mg/dL (reference range 75–105 mg/dl)

### 1.2. Blood Gas

  Capillary pH 7.29 (reference range 7.35–7.45)
  PaCO_2_ 32 mm Hg (reference range 35–45 mm·Hg)
  Base excess −10 mEq/L

### 1.3. Complete Blood Cell (CBC) Count

  White blood cells (WBC) of 18,700/*μ*L reference range (5,000–20,000/*μ*L)
  Hemoglobin of 16.7 g/dL (167 g/L) (reference 14.3–24.5 g/dl)
  Hematocrit of 53%, (reference 44–64%)
  Platelet count of 74,000/*μ*L (reference 130–450/*μ*L)

**Figure 1:**
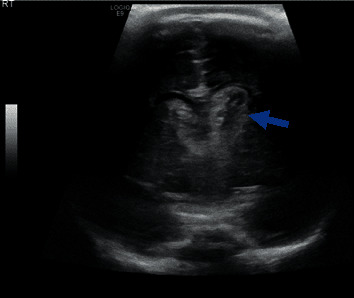
Hyperdense blood in both ventricles (left greater than right). Arrow shows left intraventricular bleeding extending into the surrounding brain parenchyma including the thalamus.

**Figure 2:**
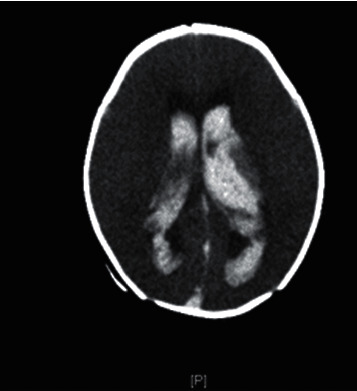
Computed tomography scan of the brain showing intraventricular hemorrhage involving the lateral third and fourth ventricles as well as the basal cistern with mild to moderate enlargement of the lateral ventricles.

There was no left shift on the CBC. In the ED, the infant received 2 normal saline boluses and was started on intravenous fluids and antibiotics and was transferred to a level IV neonatal intensive care unit for further care, where her vital signs improved. On examination, the infant was hypoactive but responsive to stimulation. She continued to have intermittent episodes of seizures (tonic-clonic seizures of both upper and lower extremity and right gaze deviation) on admission. Respirations were not labored, and heart sounds were normal on auscultation. Rest of the examination findings were within normal limits. Serum sodium concentration was 174 mEq/L, potassium 6.5 mEq/L, chloride 134 mEq/L, blood urea nitrogen 158 mg/dL, creatinine 1.61 mg/dL, and bicarbonate 15 mEq/L. Another normal saline bolus was given, and intravenous fluids continued. The WBC count was 11,800/*μ*L, and the hemoglobin and hematocrit dropped to 13.3 g/dL and 41%, respectively. The platelet count dropped significantly to 9,000/*μ*L. The infant continued to receive treatment for dehydration and sepsis. In addition, she was evaluated and treated for herpes simplex virus (HSV) infection, disseminated intravascular coagulation, and metabolic diseases. Head ultrasonography and computed tomography were performed to rule out intracranial hemorrhage because of the suspected seizure and acute drop in hemoglobin, hematocrit, and platelet count. The scans revealed significant bilateral intraventricular hemorrhage (IVH) involving the lateral, third, and fourth ventricles and basal cistern (Figures 1 and 2). In addition, a parenchymal hemorrhage was noted mainly in the ventral medial aspect of the thalamus and basal ganglia on the left side. A bedside ventricular tap was performed to relieve elevated intracranial pressure. She was given a loading dose of phenobarbital and video electroencephalography was performed, which showed that the patient continued to have electrographic seizures requiring multiple antiepileptic drug boluses of phenobarbital, levetiracetam, and fosphenytoin to stop the seizure. Further treatment included fluid rehydration with age-appropriate fluids aimed at correcting the hypernatremia by no more than 0.5 mmol/L per hour. The infant received several platelet and plasma transfusions. Electrolytes were checked every 4 to 6 hours, showing gradual improvement of levels. Also, treatment with broad-spectrum antibiotics and acyclovir was continued until sepsis and herpes simplex virus infection were ruled out. Hematologic, genetic, and metabolic testing was negative for any increased risk for bleeding or thrombosis.

**Figure 3:**
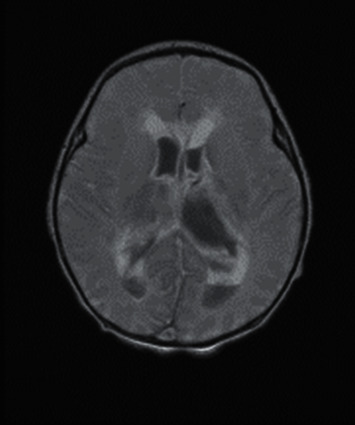
Magnetic resonance imaging of the brain showing intraventricular hemorrhage within the lateral third and fourth ventricles with hydrocephalus. Bilateral thalami and basal ganglia hemorrhage can be noted, primarily on the left side. Diffuse diffusion signal abnormality can be noted in the cerebral hemisphere, likely representing ischemic/necrotic changes.

**Figure 4:**
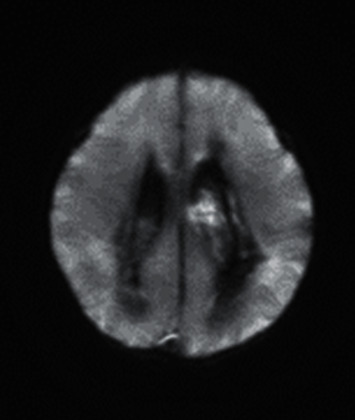
Magnetic resonance imaging of the brain showing intraventricular hemorrhage within the lateral third and fourth ventricles with hydrocephalus. Bilateral thalami and basal ganglia hemorrhage can be noted, primarily on the left side. Diffuse diffusion signal abnormality can be noted in the cerebral hemisphere, likely representing ischemic/necrotic changes.

**Figure 5:**
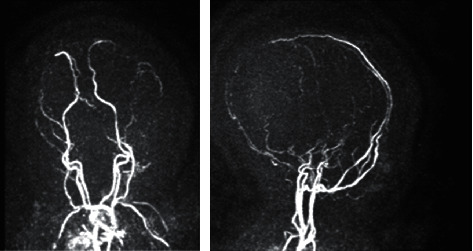
Arterial and venous magnetic resonance angiography scans of the brain showing no intracranial arterial flow. No flow signal was identified in the dural venous sinuses, likely representing underlying sinus thrombosis.

The infant's neurologic state continued to deteriorate with the cessation of spontaneous movements, absent reaction to painful stimuli, and fixed and dilated pupils. She became apneic and was intubated to support respiration. Further brain imaging with magnetic resonance imaging, including venous and arterial angiography, showed stable IVH within the lateral, third, and fourth ventricles. It also showed previously known parenchymal hemorrhages in the bilateral thalami and basal ganglia, which was more prominent on the left side. Arterial angiography showed no flow in the intracranial arteries, and venous angiography showed no flow in dural venous sinuses suggestive of cerebral venous sinus thrombosis (Figures 3–5). The infant continued to remain apneic and was unable to wean off the ventilator. After extensive counseling, the parents decided against comfort care for the infant. As per their request, tracheostomy and gastrostomy tubes were placed and the infant was discharged from the hospital.

## 2. Discussion

Breastfeeding is considered a normative feeding standard for all infants because of its extensive benefits to both the infant and mother. The American Academy of Pediatrics recommends exclusive breastfeeding for all infants until the age of 6 months, when complementary food can be added while continuing breastfeeding until 1 year of age [1]. However, some women, especially primigravida women [2] and women who have cesarean sections [3], may experience insufficient lactation or lactation failure, which leads to neonatal hypernatremic dehydration. Exclusive breastfeeding, maternal age greater than 35 years, birth gestation of less than 39 weeks, and birth hospitalization of less than 48 hours for infants born via cesarean section are also associated with an increased risk of dehydration and hypernatremia [2].

In neonates, hypernatremia is usually defined as serum sodium levels greater than 145 mEq/L (145 mmol/L) [4]. Hypernatremia is seen in most newborns with a weight loss of more than 10% from birthweight [5, 6]. It usually occurs because of either a free water deficit or excess of sodium supplementation to the neonate. Free water deficit could occur because of inadequate fluid intake such as lactation failure or inadequate lactate or from hypotonic fluid losses through the skin, respiratory system, genitourinary system, and gastrointestinal tract and in endocrine disorders such as diabetes insipidus. It can be a consequence of excess sodium supplementation such as inappropriate formula mixing or excess sodium through parenteral routes [4]. Mineralocorticoid excess can also lead to hypernatremia [7]. Hypernatremia increases extracellular osmolality, shifting intracellular fluid to extracellular space, leading to shrinkage of cells. By preserving the intravascular volume initially, signs of dehydration are less evident, leading to delayed presentation. In the brain, acute hypernatremia can cause shrinkage of the cerebral hemisphere, leading to rupture of bridging veins, resulting in intracranial hemorrhage. As an adaptive response to shrinkage, initially cells accumulate inorganic ions followed by organic osmoles. In the event of rapid correction of hypernatremia, water follows the ionic gradient into the intracellular compartment, leading to cerebral edema and central pontine myelinolysis [7–9]. When serum sodium levels reach 160 mEq/L or greater (≥160 mmol/L), this in and of itself is a risk factor for morbidity and mortality and leads to seizures, bradycardia, vascular thrombosis, disseminated intravascular coagulation, renal failure, intracranial hemorrhage, pontine myelinosis, cerebral edema, and death [7, 10]. Neonates with severe hypernatremia usually develop fever, fussiness, decreased activity, irritability, poor oral intake, poor or absent suck, jaundice, and decreased urination/defecation and in severe cases may also manifest apnea, mottling, and seizures [6, 10]. Untreated severe hypernatremia can lead to seizures, intracranial hemorrhage [11], vascular thrombosis [12], and death [13]. Boskabadi et al. followed 65 neonates admitted for mean serum sodium levels of 158 mEq/L (158 mmol/L) over 2 years and found a correlation between developmental delay at early ages and severity of hypernatremia [14]. They also found a lag in weight gain which is corrected by 12 to 18 months of age [14].

In the current case, severe hypernatremia resulted in cerebral venous sinus thrombosis, which caused significant intraventricular and intracranial hemorrhage. Severe thrombocytopenia occurred due to sinus thrombosis, which further contributed to worsening of hemorrhage. Dehydration leading to hypernatremia is one of the most common causes of cerebral venous sinus thrombosis. Other risk factors include infection, maternal chorioamnionitis, hypoxic-ischemic encephalitis, and thrombophilia [15]. The superior sagittal, lateral sinus, and straight sinuses are most commonly involved [15]. It is usually associated with IVH, periventricular congestion, and thalamic hemorrhage. Hence, it is important to consider venous sinus thrombosis in a newborn with an IVH [16]. It can usually be diagnosed using Doppler flow ultrasonography, contrast-enhanced computed tomography, or magnetic resonance venography. Currently, there is no agreement on the use of antithrombotic therapy in neonates with thrombosis with or without hemorrhage [17] and it is center dependent. In the United States, about 25% of infants with venous sinus thrombosis are treated with antithrombotics [17]. Our patient received further evaluation for alternative causes; however, testing did not reveal any nonaccidental trauma, bleeding disorders, metabolic disorders, genetic epilepsy panel findings, or meningitis. In addition, whole-exome sequencing did not reveal an alternative diagnosis.

## 3. Conclusion

Physicians should be aware of the possibility of hypernatremic dehydration in an exclusively breastfed infant. Hypernatremic dehydration in a neonate may be associated with a devastating outcome. It should be recognized early and prevented before the emergence of complications. Lactation support should be provided to ensure successful breastfeeding. Hypervigilance is key and when a newborn infant has lost more than 10% of birthweight, frequent/daily follow-up for weight checks, lactation consultation, and temporary supplementation of formula feeds may be necessary to avoid complications.

## Data Availability

The data used to support the study are available within the article.
